# Profiles of immune cell infiltration and immune-related genes in the tumor microenvironment of esophageal squamous cell carcinoma

**DOI:** 10.1186/s12920-021-00928-9

**Published:** 2021-03-10

**Authors:** Mingdi Liu, Faping Li, Bin Liu, Yongping Jian, Dan Zhang, Honglan Zhou, Yishu Wang, Zhixiang Xu

**Affiliations:** 1 Key Laboratory of Pathobiology, Ministry of Education, Jilin University, Changchun, 130021 Jilin People’s Republic of China; 2 Department of Urology, The First Hospital of Jilin University, Changchun, 130021 Jilin People’s Republic of China; 3 Division of Hematology and Oncology, Comprehensive Cancer Center, University of Alabama at Birmingham, Birmingham, AL USA

**Keywords:** Esophageal squamous cell carcinoma, Tumor microenvironment, Tumor-infiltrating immune cells, Prognosis, Overall survival

## Abstract

**Background:**

As a complex system participating in tumor development and progression, the tumor microenvironment was poorly understood in esophageal cancer especially squamous cell carcinoma (ESCC).

**Methods:**

ESTIMATE algorithm is used to investigate tumor-infiltrating immune cells and prognostic genes which were associated with the tumor microenvironment in ESCC.

**Results:**

Based on the immune and stromal scores, ESCC samples were divided into high and low score groups and 299 overlapping differentially expressed genes were identified. Functional enrichment analysis showed that these genes were mainly involved in muscle-related function. Prognostic genes including *COL9A3*, *GFRA2*, and *VSIG4* were used to establish a risk prediction model using Cox regression analyses. Then multivariate analysis showed that *COL9A3* was an independent discriminator of a better prognosis. Kaplan–Meier survival analysis showed that the expression of *COL9A3* was significantly correlated with the overall survival of ESCC patients. The area under the curve for the risk model in predicting 1- and 3- year survival rates were 0.660 and 0.942, respectively. The risk score was negatively correlated with plasma cells, while positively correlated with the proportions of activated CD4 memory T cells, M1 Macrophages and M2 Macrophages (*p* < 0.001 for each comparison). Gene set enrichment analysis suggested that both immune response and immune system process gene sets were significantly enriched in high-risk group.

**Conclusions:**

Our study provided a comprehensive understanding of the TME in ESCC patients. The establishment of the risk model is valuable for the early identification of high-risk patients to facilitate individualized treatment and improve the possibility of immunotherapy response.

**Supplementary Information:**

The online version contains supplementary material available at 10.1186/s12920-021-00928-9.

## Background

Esophageal cancer is a gastrointestinal malignancy with extremely aggressive nature and poor prognosis [1]. It is the eighth most common cancer and the sixth most common cause of cancer death globally [2]. In Iran, esophageal cancer is more popular than any other countries or regions in the world [3]. Classified by histology, esophageal cancer is divisible into adenocarcinoma and squamous cell carcinoma (ESCC) [4]. The effective methods for treatment of ESCC include chemotherapy or chemoradiotherapy followed by extensive surgery, which will obviously reduce health-related quality of life. Although recent developments have improved prognosis and survivorship, the molecular mechanism behind ESCC is not clear till now. Therefore, it is still important to identify potential biomarkers to increase the effectiveness of therapy and survival rate of ESCC patients. As an effective therapeutic option, immunotherapy, especially immune checkpoint inhibitors, shows obviously clinical benefits in various cancers [5, 6]. Instead of two well-known immune checkpoint molecules PD-1 (programmed cell death protein 1, also named CD279) and PD-L1 (programmed cell death-ligand 1), other molecules such as CD155, CD226, and LAG3 are also recognized as new immune-related molecules which contribute to tumor-mediated immune suppression and promote tumor immunity escape in ESCC [7]. Tumor-infiltrating immune cells (TIICs), as the main components of the tumor microenvironment (TME) which composed of stromal cells, endothelial cells, and TIICs [8], have a significant impact on tumor progression, treatment, and outcomes of patients. Recently, more researches have paid attention to antitumor immunity regulated by immune microenvironment [9–11], but the mechanism regulating the infiltration of immunocytes in ESCC is poorly understood. Thus, identifying the TME related therapeutic targets may improve immunotherapy efficacy and give a new clue for clinical strategy. Based on DNA copy number, ESTIMATE (Estimation of Stromal and Immune cells in Malignant Tumor tissues using Expression data) uses gene expression signatures to infer the fraction of stromal and immune cells in different tumor samples. Immune scores and stromal scores were calculated to predict the level of infiltrating stromal and immune cells to infer tumor purity in tumor tissue, while samples with low tumor purity showed high stromal and immune scores [12]. The researches in pancreatic adenocarcinoma, lung cancer, glioblastoma, osteosarcoma, and other types of cancers [13–16] showed that this newly developed method is reliable for molecule screen.

In this current study, we first calculated immune and stromal scores of 81 ESCC tissues in the TCGA database using ESTIMATE algorithm and then retrieved immune-associated differentially expressed genes (DEGs). Then, the correlation between immune/stromal scores and clinical characteristics, prognosis of ESCC patients including age(years), gender, pathologic TNM tumor stage, and tumor grade were analyzed respectively. A predictive risk model to estimate patient outcome was established and the associations of the TME-related risk score with the levels of TIICs and immune pathways were analyzed.

## Methods

### Dataset and estimation of stromal and immune scores

The microarray studies of ESCC analyzed during the current study were available in The Cancer Genome Atlas (TCGA) (dataset ID: TCGA-ESCA, https://gdc.xenahubs.net/download/TCGA-ESCA.htseq_counts.tsv.gz). Both the gene expression and clinical data used in this research are publicly available and classified as open-access. To evaluate the infiltrating levels of the immune and stromal cells in the ESCC tissues, “estimate” R package (version 1.0.13) was used as a tool of ESTIMATE algorithm for calculation of immune and stromal scores. Based on the median value of immune/stromal scores, the ESCC patients were divided into high and low score groups to identify a possible association of these scores with overall survival.

### Identification of DEGs based on immune and stromal scores

We used the “limma” R package (version 3.42.2) to identify the DEGs between high and low score groups. Log2FC > 1.5 or log2FC < (− 1.5) and *p* < 0.05 were set as the threshold for genes screening both in immune scores group and stromal scores.

### Functional enrichment analysis

Then, the functional enrichment analyses of Gene Ontology (GO) and Kyoto Encyclopedia of Genes and Genomes (KEGG) pathway were analyzed using “clusterProfiler” package (version 3.14.3), and *p* < 0.05 was considered to indicate statistical significance.

### Establishment of TME-related risk model and survival analysis

Using the “survival” R package (version 3.1-11), the univariate and multivariate Cox regression analyses were performed for analyzing associations of the levels of DEGs with overall survival. Consequently, powerful prognostic genes were screened out and then risk scores were calculated for all ESCC patients. Based on the median of risk scores, all patients were divided into high- (n = 41) and low- risk (n = 40) group. We used Kaplan–Meier curves to exhibit the association between risk score and overall survival. The log-rank test was employed to test the statistic difference with the significance level *p* < 0.05. Using the “timeROC” package (version 0.4), the survival receiver operating characteristic curve (ROC) with the area under the curve (AUC) value was visualized.

Gene set enrichment analysis (GSEA) was carried out to evaluate associations of immune pathways with the TME-related risk score. *p* < 0.05 indicated statistical significance.

### Association between risk scores and the levels of TIICs

The online analytical platform CIBERSORT with an arrangement of 1000 default statistical parameter was used to quantify the relative proportions of 22 TIICs in the TME of ESCC. Wilcoxon rank-sum test was used to determine the association between risk scores and the differential proportions of 22 TIICs in ESCC tissues. *p* < 0.05 was considered as statistical significance. Spearman rank analysis was performed using GraphPad Prism version 8.0.0 for Windows (GraphPad Software, San Diego, California USA, www.graphpad.com).

## Results

### The immune and stromal scores were tightly associated with tumor grade.

**Fig. 1:**
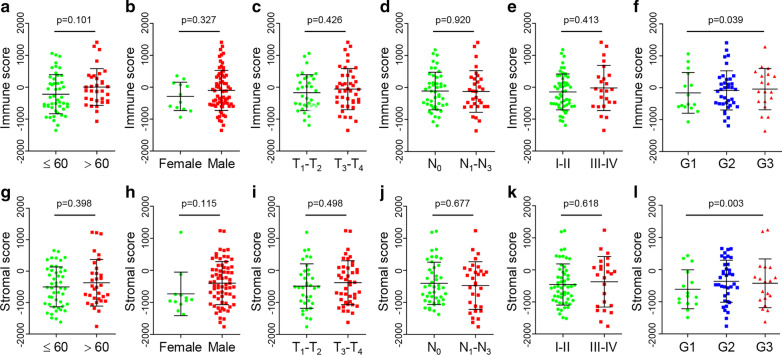
Associations of immune and stromal scores with ESCC clinicopathological characteristics. **a**, **g** Distributions of immune and stromal scores among different ages, **b, h** gender, **c**–**e**, **i**–**k** tumor stage (TNM), and **f, l** tumor grade. ESCC, esophageal squamous cell carcinoma

Using the ESTIMATE algorithm, we first determined immune and stromal scores of 81 ESCC samples based on the gene expression data obtained from the TCGA database (Additional File 1: Table S1). Statistical analysis showed that age, gender, and TNM stage had no correlation with both immune score (Fig. 1a–e) and stromal score (Fig. 1g–k). But high-grade tumors (G2-G3) had higher immune and stromal scores than G1 tumors (*p* = 0.039 and 0.003, respectively), while G2 tumor had the highest immune score and G3 tumor had the highest stromal score (Fig. 1f, l).

**Fig. 2:**
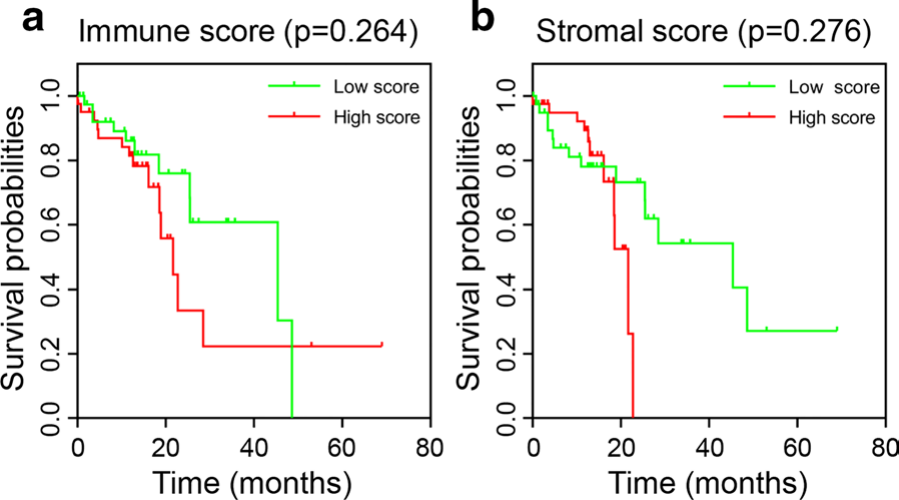
Kaplan–Meier survival curves of ESCC patients with low vs. high immune scores (**a**) and stromal scores (**b**)

Based on the median of immune and stromal scores of every sample, we divided 81 samples into two groups, that is, 41 samples were in the high-score group while 40 in the low-score group. Kaplan–Meier survival curves revealed that no significant results were found neither in immune score groups or stromal score groups (*p* = 0.264 and 0.276, Fig. 2a, b).

### Identification of differentially expressed genes between high and low immune/stromal scores

**Fig. 3:**
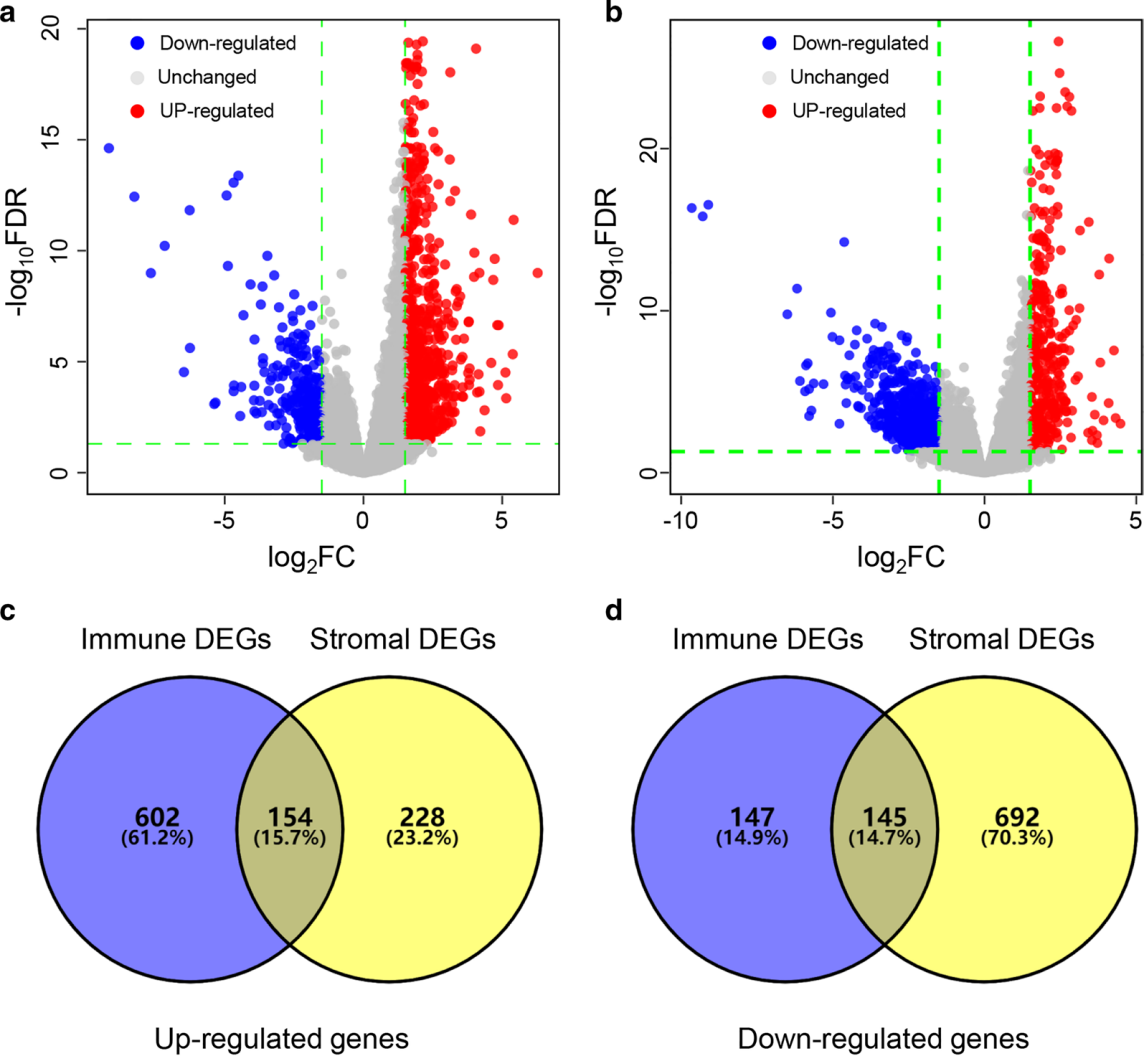
Identification of differentially expressed genes. **a**–**b** Volcano plot of DEGs based on immune and stromal score in ESCC samples. **c**–**d** Venn diagrams showing the overlapping genes among high and low immune/stromal DEG, respectively. DEGs, differentially expressed genes; ESCC, esophageal squamous cell carcinoma

To identify the immune-related and stromal-related genes, we performed differential analysis using “limma” packages (version 3.42.2) in R (version 3.6.1) based on genes expression level. As shown in volcano plots, there were a total of 1048 and 1219 TME-related DEGs between high and low immune/stromal score groups, respectively (Fig. 3a, b). Compared with the low score group, there were 756 up-regulated and 292 down-regulated genes in the high immune score group, while 382 up-regulated and 837 down-regulated genes in the high stromal score group (Fig. 3c, d). Subsequently, 299 overlapping genes in Venn diagrams were selected for further analysis.

### Functional enrichment analysis

**Fig. 4:**
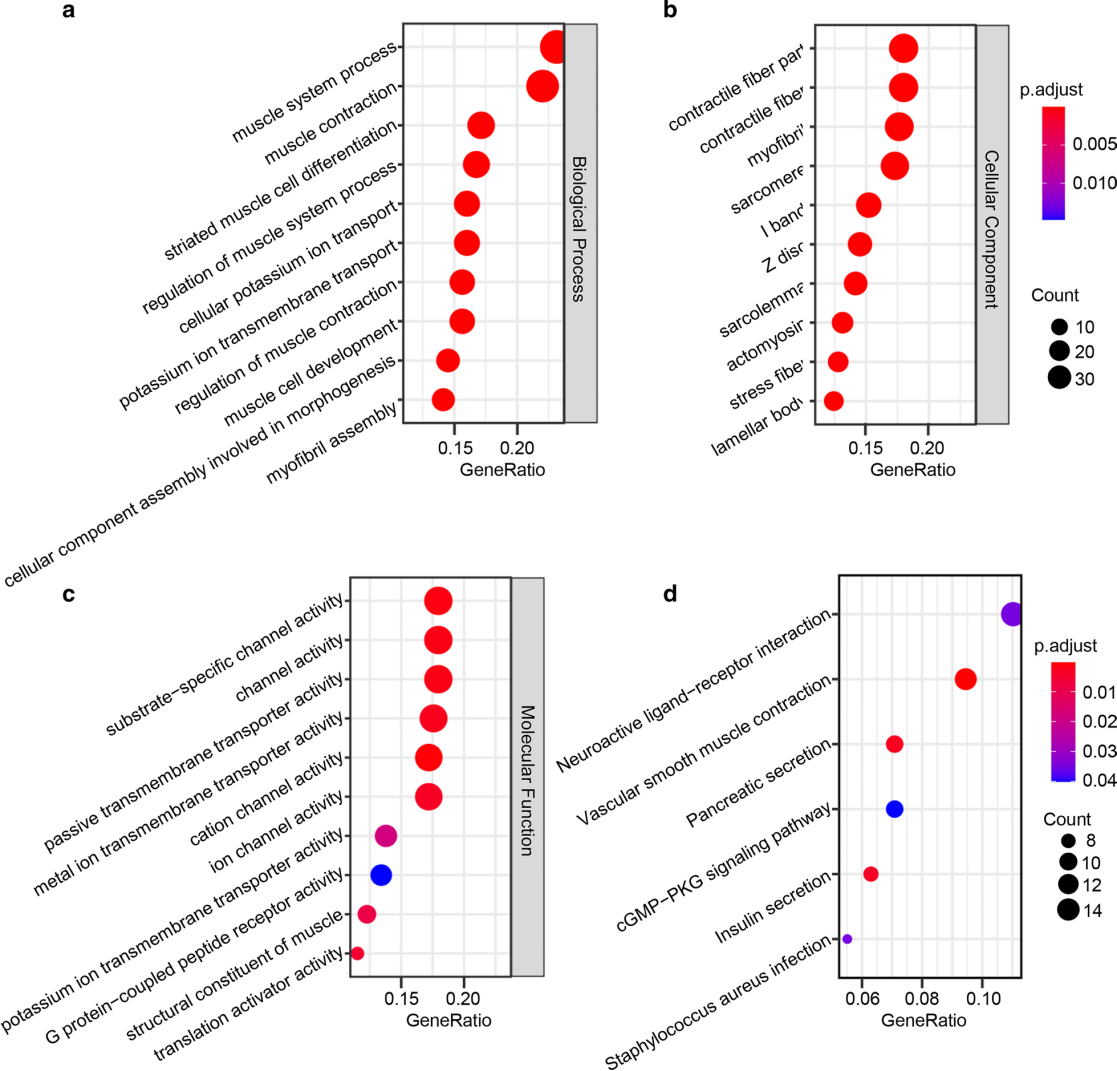
Enrichment analysis of microenvironment related differentially expressed genes. **a**–**c** The top 10 of biological processes, cellular component and molecular function GO terms, respectively. **d** The top 10 enriched KEGG pathways. GO, Gene Ontology; KEGG, Kyoto Encyclopedia of Genes and Genomes

Functional enrichment analysis was performed to comprehend the functional properties of the 299 TME-related DEGs using the “clusterProfiler” package (version 3.14.3) in R (version 3.14.3). We selected the top 10 GO terms in each biological process (Fig. 4a), cellular component (Fig. 4b), and molecular function (Fig. 4c). The results showed that these DEGs mainly enriched in the biological process including muscle system process, muscle contraction, and striated muscle cell differentiation. Similarly, contractile fiber part, contractile fiber, and myofibril which mainly related to muscle structure were most enriched by selected DEGs. As for molecular function, substrate-specific channel activity, channel activity, and passive transmembrane transporter activity were mainly annotated. In the KEGG pathway annotation and enrichment analysis, we found that enriched pathways were associated with neuroactive ligand-receptor interaction, vascular smooth muscle contraction, and pancreatic secretion (Fig. 4d).

### Establishment of the risk prediction model

**Fig. 5:**
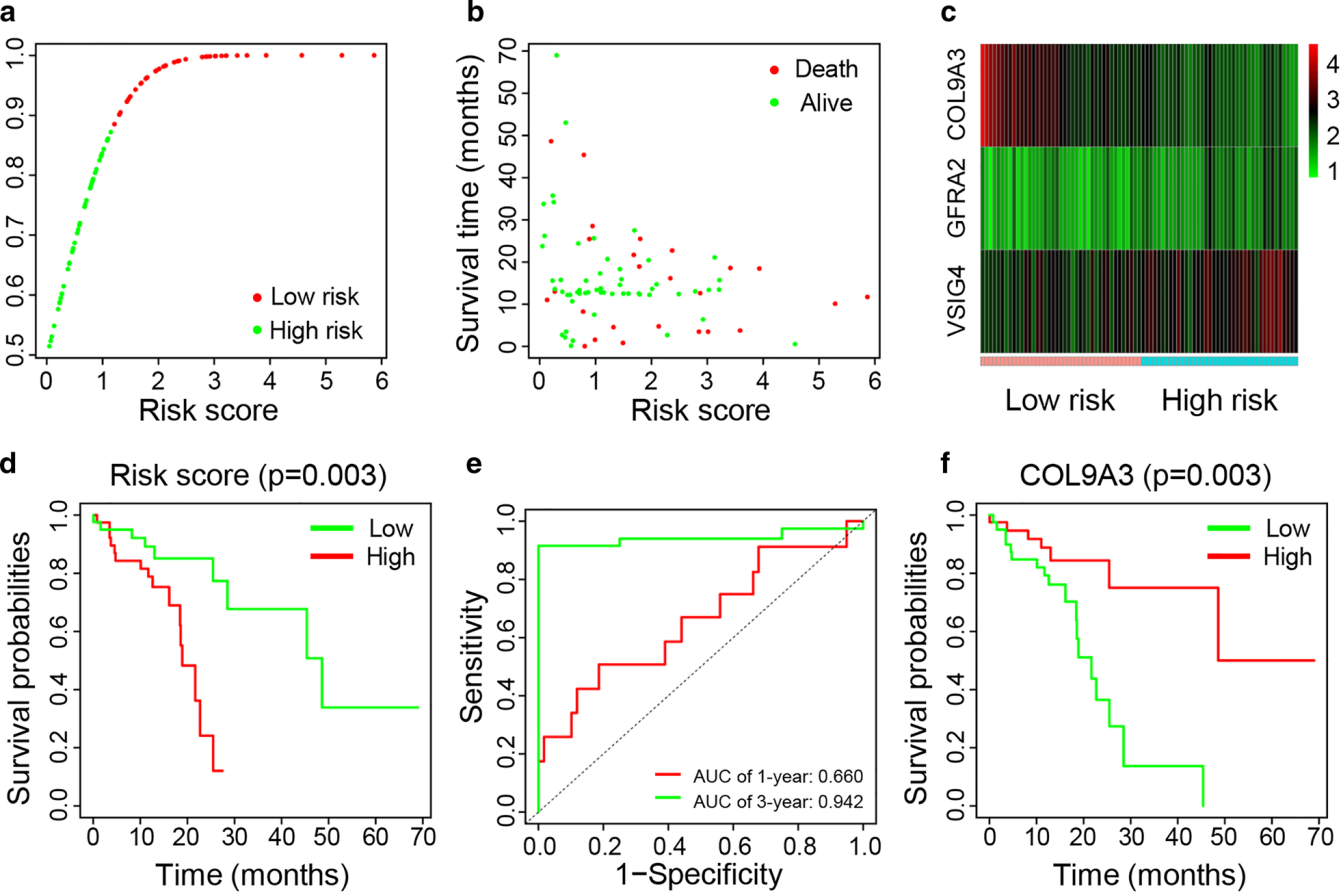
Establishment and assessment of the predictive risk model. **a** Dot plot of risk score. Horizontal and vertical axes respectively represent risk score and ESCC samples, ranked by increasing risk score. Red and green colors represent high- and low- risk cases, respectively. **b** Dot plot of survival. Red and green colors represent dead and living ESCC cases, respectively. **c** Heat map of the expression levels of the three genes. Vertical and horizontal axes respectively represent genes and ESCC samples, ranked by increasing risk score. Genes with higher, lower, and same expression levels are shown in red, green, and black, respectively. Color bars at the bottom of the heat map represent sample types, with pink and blue indicating low- and high-risk score samples, respectively. **d** Overall survival curves obtained by the Kaplan–Meier method. P-values were obtained by the log rank test. **e** ROC curves of the risk model for predicting 1- and 3-year survival rates. **f** Kaplan–Meier curves of CSS for ESCC patients grouped by expression levels of COL9A3. ESCC, esophageal squamous cell carcinoma; ROC, receiver operating characteristic; CSS, cancer-specific survival; COLPA3, collagen type IX alpha 3 chain

According to the results of univariate Cox regression analysis of prognostic factors, *COL9A3*, *VSIG4* and *GFRA2* were significantly associated with overall survival (*p* < 0.05 for each comparison). And multivariate Cox regression results showed that *COL9A3* was an independent prognosis factor for ESCC patients (*p* = 0.003) (Table 1). These results suggested their expression level in ESCC patients were possibly related to the prognosis of ESCC. As a result, we selected and used *COL9A3*, *VSIG4* and *GFRA2*, which were significantly associated with overall survival in ESCC, to build a risk model to give some clinical information related to patients’ outcomes for further exploration. Constantly, the risk score was obtained based on relative coefficients in Cox regression according to the formula: (− 0.3937 * *COL9A3* expression level) + (0.1902 * *GFRA2* expression level) + (0.1441 * *VSIG4* expression level) (Additional File 1: Table S2). The median value of risk scores was used as a cutoff to divide samples into high- and low-risk groups for further study (Fig. 5a). As shown in the scatter plot, patients with lower risk scores showed higher survival probability than those with higher risk scores (Fig. 5b). Moreover, compared with the low-risk group, higher expression levels of *GFRA2* and *VSIG4* were observed in the high-risk group. Conversely, the cases with the higher expression level of *COL9A3* were in the low-risk group, confirming that *COL9A3* is a good prognostic factor (Fig. 5c). The Kaplan–Meier survival analysis revealed that high-risk score was significantly associated with a poor outcome (Fig. 5d). As for ROC curve, the AUC for the risk model in predicting 1- and 3- year survival rates were 0.660 and 0.942, respectively (Fig. 5e), which probably indicated the risk model we established may have a good predictability for patients’ outcome. Moreover, the survival curve showed that a high *COL9A3* expression level was related to low survival probabilities (Fig. 5f).

**Table 1:** Univariate and multivariate Cox regression analysis of prognostic factors for overall survival

Variables	Univariate analysis		Multivariate analysis	
	HR (95% CI)	*p* value	HR (95% CI)	*p* value
*COL9A3*	0.689(0.538–0.881)	**0.003**	0.675(0.519–0.877)	**0.003**
*VSIG4*	1.321(1.008–1.732)	**0.044**	1.155(0.782–1.705)	0.468
*GFRA2*	1.331(1.007–1.759)	**0.045**	1.210(0.798–1.832)	0.370

*HR* Hazard ratio, *95% CI* 95% confidence interval

The significant values (*p* < 0.05) are marked in bold

### The relationship between immune scores and TIICs

**Fig. 6:**
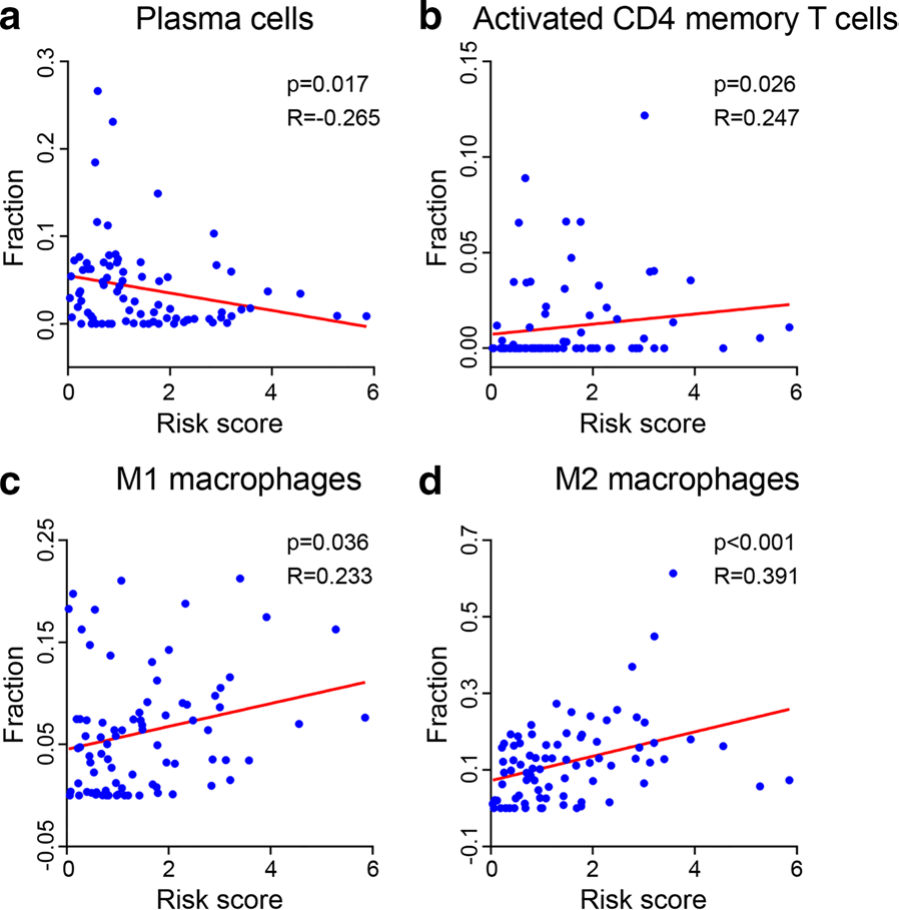
The association between risk score and the levels of 4 TIICs in ESCC tissues analyzed by Spearman rank analysis, including plasma cells (**a**), T cells CD4 memory activated (**b**), M1 (**c**) and M2 (**d**) macrophages

Using spearman rank analysis in GraphPad Prism 8.0, we identify the association between risk score and the infiltration levels of 22 TIICs in ESCC tissues (Table 2). The results showed that the risk score was negatively correlated with the proportions of plasma cells (r = − 0.265, *p* = 0.017) (Fig. 6a) while positively correlated with the proportions of activated CD4 memory T cells (r = 0.247, *p* = 0.026), M1 Macrophages (r = 0.233, *p* = 0.036) and M2 Macrophages (r = 0.391, *p* < 0.001) (Fig. 6b–d).

**Table 2:** Spearman rank analysis to determine the association between risk score and the levels of 22 TIICs in ESCC tissues

Tumor-infiltrating immune cell	Risk score	
	Spearman r	*p* value
B cells memory	0.091	0.417
B cells naive	− 0.164	0.143
Dendritic cells activated	− 0.047	0.674
Dendritic cells resting	0.127	0.259
Eosinophils	− 0.039	0.730
Macrophages M0	− 0.159	0.157
Macrophages M1	0.233	**0.036**
Macrophages M2	0.391	** < 0.001**
Mast cells activated	− 0.172	0.124
Mast cells resting	0.188	0.093
Monocytes	− 0.021	0.852
Neutrophils	0.088	0.436
NK cells activated	0.057	0.616
NK cells resting	− 0.046	0.686
Plasma cells	− 0.265	**0.017**
T cells CD4 memory activated	0.247	**0.026**
T cells CD4 memory resting	− 0.178	0.112
T cells CD4 naive	− 0.098	0.384
T cells CD8	0.105	0.349
T cells follicular helper	− 0.114	0.311
T cells gamma delta	− 0.014	0.899
T cells regulatory (Tregs)	− 0.033	0.771

The significant values (*p* < 0.05) are marked in bold

### The involvement of immune pathways predicted by risk model

**Fig. 7:**
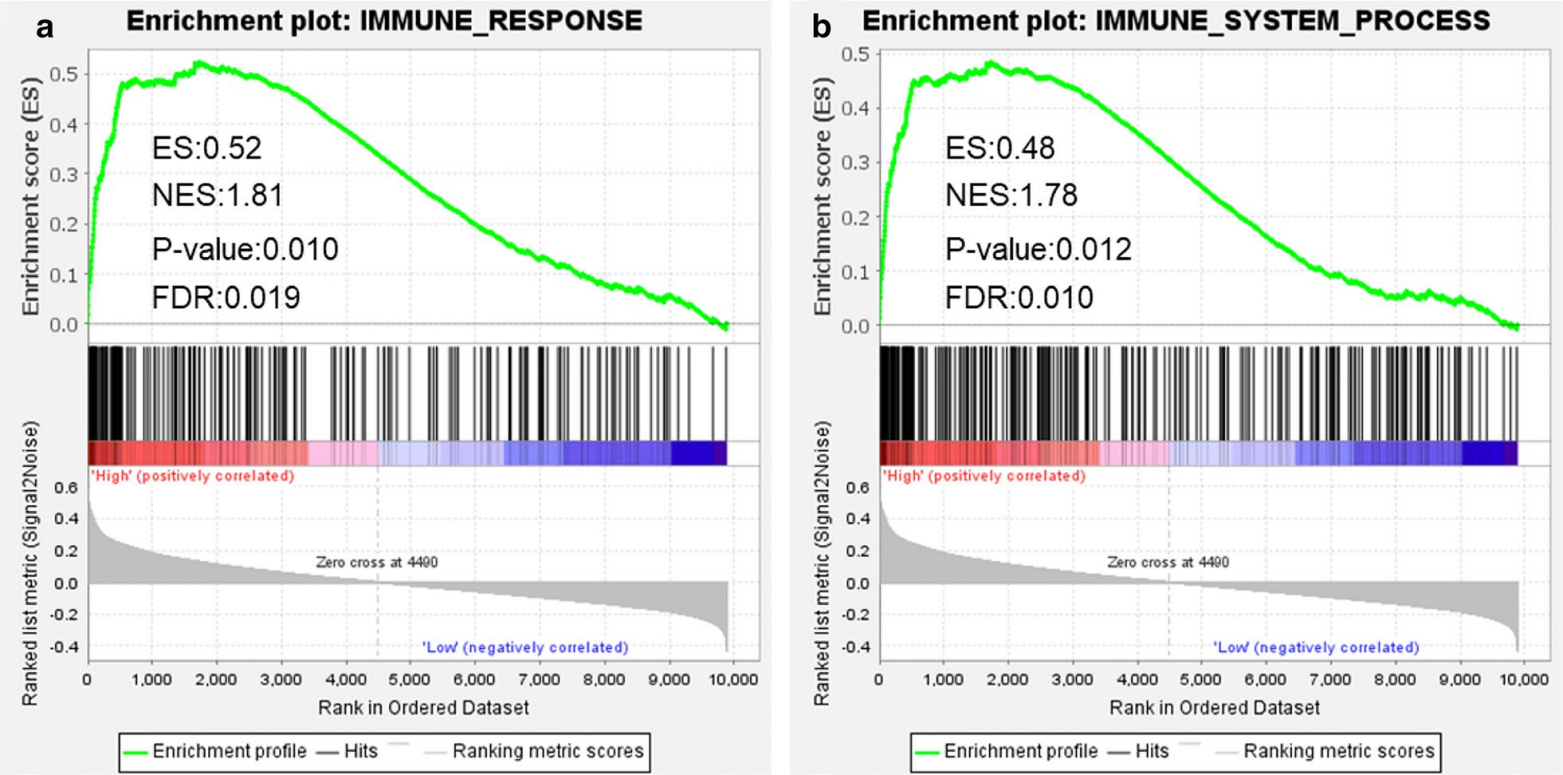
GSEA of the risk score in ESCC. The horizontal axis represents genes of the immune response (**a**) and immune system process (**b**) gene sets, ranked by decreasing risk score. The vertical axis represents enrichment score. ESCC, esophageal squamous cell carcinoma; GSEA, gene set enrichment analysis; ES, enrichment score; NES, normalized enrichment score

With online database Molecular Signatures Databases v4.0 (http://software.broadinstitute.org/gsea/downloads.jsp), we retrieved two immune gene sets, involving immune response (M19817) and immune system process (M13664). GSEA results showed that both immune response and immune system process gene sets were significantly enriched in the high-risk group (*p* = 0.010 and 0.012, respectively) (Fig. 7a, b).

## Discussion

TME is one of the main hallmarks of cancer, so it is important to identify the key druggable factors and pathways in the TME. The response to cancer immunotherapy especially immune checkpoint inhibitors were impacted by tumor immune microenvironment. Wang et al. found that an “infiltrated-excluded” or “cole” tumor immune microenvironment is predictive of poor response and low-dose metformin reprograms the TME in ESCC [17]. Research by Strizova et al. showed that FasR^+^ NK cells, CD4^+^ , and CD8^+^ T cells infiltrated lymph nodes at the lowest levels and that the FasR^+^ DR3^+^ CD4^+^ T cells were enhanced in esophageal cancer [18]. These compartmental proportions correlated with tumor stage and tumor grade suggested new possibilities for personalized immunotherapy for patients.

Therefore, in the current study, we conducted the bioinformatics analysis of TIICs and TME-related genes in ESCC and established contacts with the clinical outcome and prognosis of ESCC patients for potential prognostic biomarker selection. ESTIMATE, as a common method for calculation of immune and stromal scores, can bring scientific evidence to further analysis. We first obtained 81 ESCC samples with clinical data from the TCGA database. Then, using the ESTIMATE algorithm, we calculated immune and stromal scores of these patients. The correlation between these scores and clinical characteristics were also analyzed. As a result, there was a significance between immune and stromal scores and tumor grades. It is suggested that the tumor immune microenvironment in ESCC had a potential influence on tumor differentiation. Besides, identifying the biomarkers related to TME may predict and even improve the prognosis of ESCC patients.

Continuously, 299 TME-related DEGs were obtained between the high and low immune/stromal score groups. GO annotation results showed that these genes were not enriched in immune-related signaling but almost be relative to muscle function and structure, which indicated a new clue for ESCC development. Skeletal muscles contain resident immune cells and there is a cross-talk between muscle and innate immune cells in physiological and pathogenic conditions, including inflammatory myopathies, endotoxemia, or different types of muscle injury/insult [19]. Paracrine/autocrine and contact interactions have been proven to be involved in these pathological events [19]. In addition, innate immune receptors such as toll-like receptors and NOD-like receptors have influences on skeletal muscle metabolism and the muscle cells have the ability to secrete factors affecting the immune system [20]. These findings showed the correlation between immune response and muscle physiological effect, but there was little research followed with interest of tumor genesis. In our study, 299 TME-related DEGs were mainly involved in muscle system process, muscle contraction, and striated muscle cell differentiation in biological process enrichment analysis. And contractile fiber part, contractile fiber, and myofibril which mainly related to muscle structure were most enriched in cellular component. In molecular function enrichment, substrate-specific channel activity, channel activity and passive transmembrane transporter activity were 3 most significant signal in selected DEGs. In the enrichment analysis of KEGG pathways, we found that only six pathways had significantly statistics including neuroactive ligand-receptor interaction, vascular smooth muscle contraction, pancreatic secretion, cGMP-PKG signaling pathway, insulin secretion and staphylococcus aureus infection. These results indicated that the TME-related genes were involved in not only tumor immune microenvironment but also other undiscovered relative signal pathways.

Based on univariate and multivariate Cox regression analyses, three prognostic genes were identified and used to establish a risk model for predicting the prognosis of ESCC patients. The AUC value of 3-year survival was infinitely close to 1, which indicated a strong capability for predicting survival in ESCC patients. Among these three genes, *COL9A3* was identified as an independent prognosis factor in ESCC. And its expression was positively correlated with the clinical outcome, that is, the patients with high expression level of *COL9A3* has longer survival time than low expression group (*p* = 0.003).

*COL9A3* encodes the major collagen component of hyaline cartilage, which is one of the three alpha chains of type IX collagen. Type IX collagen, a heterotrimeric molecule, was usually found in tissues containing type II collagen, a fibrillar collagen [21]. Mutations in this gene were usually found in the patients with multiple epiphyseal dysplasia type 3. Previous study has proven that the allelic variants in the collagen IX gene-*COL9A3* was a genetic risk factor for intervertebral disc disease [22], and two single nucleotide polymorphisms introducing in *COL9A3* were linked to an increased risk of lumbar disc disease [23]. In addition, in X-linked adrenoleukodystrophy patients, the combination of methylation levels of *SPG20*, *UNC45A*, and *COL9A3* and also the expression levels of *ID4* and *MYRF* would be a good marker for distinguishing the discriminating childhood from adult inflammatory phenotypes [24]. As for tumor-related research, *COL9A3* was identified as tumor suppressor gene in rectal cancer [25], and it was also significantly associated with the prognosis of triple-negative breast cancer as an independent prognostic signature [26]. Seldom research about the relationship between *COL9A3* and ESCC was taken by now.

*GFRA2* named Glial cell line-derived neurotrophic factor family receptor alpha 2. In human neuroblastoma cells and tissues, *GFRA2* was upregulated. It can promote cell proliferation by interacting with the tumor suppressor PTEN in neuroblastoma [27]. Similarly, a high expression level of *GFRA2* leads to PTEN inactivation and then promotes tumor cell growth and chemoresistance in pancreatic cancer [28, 29]. It is suggested that *GFRA2* may have the same effects on ESCC, as another tumor type in the digestive system. But the specific relationship between *GFRA2* expression and ESCC development deserves further test and verification.

*VSIG4* encodes a protein that may be a negative regulator of T-cell response. It broadly expressed in placenta, lung, and 19 other tissues. Byun et al. showed that high *VSIG4* expression of cancer tissue was associated with a longer disease-free interval in benign ovarian tumors [30] and hepatitis B virus-related hepatocellular carcinoma [31]. Both Xu et al. and Hu et al. found that *VISG4* could be used as a prognostic factor and a potential immunotherapeutic target for glioma [32] and clear cell renal cell carcinoma [33]. Similarly, Waldera-Lupa et al. found that together with other 2 genes, *VSIG4* could be a novel biomarker for supporting the diagnosis of primary central nervous system lymphomas [34]. Tumor-associated macrophage is the prominent component of lung cancer stroma and *VSIG4* may play a cancer-promoting effect in lung carcinoma development [35]. In summary, specific targeting of *VSIG4* may prove to be an efficacious strategy for the treatment of ESCC, but more research should be taken for further investigation.

Plasma cell was derived from small B lymphocytes after their activation and related with some important process in tumor progression. It showed that tumor-associated plasma cell signatures emerged as a significant signal of survival for diverse solid tumors, but its infiltrated levels was associated with poor prognosis of patients both in breast and lung adenocarcinomas [36]. In triple-negative breast cancers, the infiltration level of plasma cells was highly connected with the disease recurrence [37]. With context-dependent immune responses influenced by oncogenic drivers and the presence of inflammation, CD4^+^ T cells carried complex and important roles within tumor microenvironments [38]. Tumor-associated macrophages are heterogeneous with diverse functions. For example, M1 macrophages inhibit tumor growth, as M2 macrophages promote tumor growth. And their phenotype and functions are regulated by the surrounding micro-environment especially TME. Due to the key roles in tumor progression, cell invasion, and metastasis [39], direct targeting tumor-related macrophages may be a potential therapy strategy for patients. These results indicated that the infiltration level may have potential significance in ESCC. Using spearman rank analysis, we found that the risk score calculated by risk model was negatively correlated with the proportions of plasma cells and positively correlated with the proportions of activated CD4 memory T cells, M1 Macrophages and M2 Macrophages. It is suggested that ESCC patients with high infiltration level in activated CD4 memory T cells, M1 Macrophages and M2 Macrophages need more attention in clinical therapy. In contrast, patients with high infiltration level in plasma cells may have better prognosis and more survival time than other patients.

Finally, GSEA was performed and confirmed the close relationship between the risk scores and immune pathways. As it shown that the pathways significantly enriched in the high-risk group involved immune response and immune system process, suggesting that immunosuppression exists in high-risk ESCC patients and these high-risk patients may have a poor outcome due to un-worked immune response.

## Conclusion

In conclusion, our study provided a comprehensive understanding of the TME and identified a list of TME-related prognostic genes for ESCC patients. The establishment of the risk model is valuable for the early identification of high-risk patients to facilitate individualized treatment and improve the possibility of immunotherapy response.

## Supplementary Information

**Additional file 1: Supplementary Table 1**. The immune and stromal scores for 81 ESCC samples from TCGA database. **Supplementary Table 2**. The risk scores for 81 ESCC samples from TCGA database.

## Data Availability

The dataset downloaded from TCGA and used in this study can be found at https://gdc.xenahubs.net/download/TCGA-ESCA.htseq_counts.tsv.gz. Accession numbers for datasets in TCGA can also be found in Additional File 1: S1 and S2.

## Abbreviations

ESCC: Esophageal squamous cell carcinoma
PD-1: Programmed cell death protein 1
PD-L1: Programmed cell death-ligand 1
TIICs: Tumor-infiltrating immune cells
TME: Tumor microenvironment
ESTIMATE: Estimation of Stromal and Immune cells in Malignant Tumor tissues using Expression data
DEGs: Differentially expressed genes
GO: Gene ontology
KEGG: Kyoto Encyclopedia of Genes and Genomes
ROC: Receiver operating characteristic curve
AUC: Area under the curve
GSEA: Gene set enrichment analysis
