# Supplementary material for: Profiles of immune cell infiltration and immune-related genes in the tumor microenvironment of esophageal squamous cell carcinoma
Source: BMC Med Genomics. 2021 Mar 10;14:75. doi: 10.1186/s12920-021-00928-9 (PMC7944628; doi:10.1186/s12920-021-00928-9)
Supplement: Supplementary file 1 — Additional file 1: Supplementary Table 1. The immune and stromal scores for 81 ESCC samples from TCGA database. Supplementary Table 2. The risk scores for 81 ESCC samples from TCGA database. [file 12920_2021_928_MOESM1_ESM.pdf]

Supplementary Table 1 The immune and stromal scores for 81 ESCC samples from TCGA database.

Sample ID	Stromal score	Immune score	Sample ID	Stromal score	Immune score
TCGA-IC-A6RF-01	-954.576	27.33379	TCGA-LN-A4A8-01	155.3377	-50.7081
TCGA-IG-A3I8-01	-977.247	-940.142	TCGA-LN-A4A9-01	347.2253	1060.14
TCGA-IG-A3QL-01	-540.409	-1195.31	TCGA-LN-A4MQ-01	-1115.98	-1352.42
TCGA-IG-A3YA-01	654.9042	1011.989	TCGA-LN-A5U5-01	-1140.75	-50.7398
TCGA-IG-A3YB-01	-63.8469	373.2683	TCGA-LN-A5U6-01	-20.6301	-401.298
TCGA-IG-A3YC-01	519.3484	1408.792	TCGA-LN-A5U7-01	-829.058	-519.047
TCGA-IG-A4P3-01	-538.764	969.0434	TCGA-LN-A7HV-01	-1030.9	-526.251
TCGA-IG-A50L-01	161.323	-313.183	TCGA-LN-A7HW-01	415.5533	-809.651
TCGA-IG-A51D-01	-131.488	270.6585	TCGA-LN-A7HX-01	-472.871	2.266263
TCGA-IG-A5B8-01	-1072.16	52.31335	TCGA-LN-A7HY-01	365.0651	107.6909
TCGA-IG-A5S3-01	-749.009	-472.044	TCGA-LN-A7HZ-01	17.24853	764.9932
TCGA-IG-A625-01	-448.27	-360.137	TCGA-LN-A8HZ-01	-1228.09	-915.637
TCGA-IG-A6QS-01	-400.489	446.5135	TCGA-LN-A8I0-01	-519.553	-501.492
TCGA-IG-A8O2-01	-1272.08	-120.426	TCGA-LN-A8I1-01	-944.784	357.5318
TCGA-IG-A97H-01	-353.997	369.1213	TCGA-LN-A9FO-01	-818.31	371.8126
TCGA-IG-A97I-01	-472.544	-278.603	TCGA-LN-A9FP-01	1187.105	131.8274
TCGA-JY-A6FA-01	-1518.6	-1044.75	TCGA-LN-A9FQ-01	88.43961	-425.265
TCGA-JY-A6FD-01	-913.08	76.21551	TCGA-LN-A9FR-01	646.8421	16.47184
TCGA-JY-A6FE-01	69.14853	654.438	TCGA-S8-A6BW-01	434.3177	-558.12
TCGA-JY-A93F-01	-1263.02	-743.691	TCGA-V5-A7RC-01	-686.854	-880.172
TCGA-KH-A6WC-01	-1181.8	-350.313	TCGA-V5-A7RC-06	-1050.47	-537.738
TCGA-L5-A43J-01	-288.717	-116.059	TCGA-V5-AASV-01	-808.976	506.2377
TCGA-L5-A4OM-01	-1374.6	-685.507	TCGA-VR-A8EO-01	-677.083	-315.021
TCGA-L5-A88S-01	1222.061	318.5368	TCGA-VR-A8EP-01	-1621.44	-918.83
TCGA-L5-A88W-01	-702.858	-381.511	TCGA-VR-A8ER-01	-53.9624	-68.4265
TCGA-L5-A88Z-01	-769.809	-595.462	TCGA-VR-A8ET-01	-1024.79	-556.089
TCGA-L5-A8NK-01	-859.652	-227.073	TCGA-VR-A8EU-01	-247.353	321.1479
TCGA-L5-A8NQ-01	-246.79	423.7579	TCGA-VR-A8EW-01	-1335.34	-597.431
TCGA-L7-A56G-01	-886.025	-427.9	TCGA-VR-A8EX-01	-1760.09	-110.146
TCGA-LN-A49M-01	-515.101	-601.282	TCGA-VR-A8EY-01	-1058.59	-637.175
TCGA-LN-A49O-01	-372.807	610.5149	TCGA-VR-A8EZ-01	-156.326	-468.005
TCGA-LN-A49P-01	374.8545	1178.742	TCGA-VR-A8Q7-01	-741.199	171.9456
TCGA-LN-A49S-01	-1173.9	6.131202	TCGA-VR-AA4G-01	-125.35	259.6771
TCGA-LN-A49U-01	-73.5141	-1069.11	TCGA-VR-AA7I-01	266.5434	818.5498
TCGA-LN-A49W-01	1236.057	1283.108	TCGA-XP-A8T6-01	-561.816	-507.543
TCGA-LN-A49X-01	422.9032	119.4954	TCGA-XP-A8T8-01	-1491.37	-592.113
TCGA-LN-A49Y-01	-569.832	-757.191	TCGA-Z6-A8JD-01	-177.646	112.4839
TCGA-LN-A4A1-01	-229.458	-133.009	TCGA-Z6-A8JE-01	108.1366	-595.48
TCGA-LN-A4A3-01	-999.729	-473.362	TCGA-Z6-A9VB-01	-1267.55	-613.213
TCGA-LN-A4A4-01	20.97658	-948.078	TCGA-Z6-AAPN-01	-729.126	614.4526
TCGA-LN-A4A5-01	621.8989	148.6358			

Supplementary Table 2 The risk scores for 81 ESCC samples from TCGA database.

Sample ID	Risk score	Sample ID	Risk score
TCGA-IG-A3I8-01A	0.045181	TCGA-IC-A6RF-01A	1.197931
TCGA-IG-A3QL-01A	0.269481	TCGA-IG-A3YA-01A	2.706942
TCGA-IG-A51D-01A	0.792244	TCGA-IG-A3YB-01A	1.992808
TCGA-IG-A5S3-01A	0.030502	TCGA-IG-A3YC-01A	1.706571
TCGA-IG-A625-01A	0.274677	TCGA-IG-A4P3-01A	1.50625
TCGA-JY-A6FA-01A	0.50274	TCGA-IG-A50L-01A	3.535086
TCGA-JY-A6FD-01A	0.241623	TCGA-IG-A5B8-01A	1.854472
TCGA-JY-A93F-01A	0.553017	TCGA-IG-A6QS-01A	5.010349
TCGA-L5-A43J-01A	0.579291	TCGA-IG-A8O2-01A	2.205108
TCGA-L5-A4OM-01A	0.207236	TCGA-IG-A97H-01A	1.639502
TCGA-L5-A88W-01A	1.101454	TCGA-IG-A97I-01A	1.341092
TCGA-L5-A8NQ-01A	1.026028	TCGA-JY-A6FE-01A	4.256999
TCGA-L7-A56G-01A	0.084983	TCGA-KH-A6WC-01A	2.435362
TCGA-LN-A49S-01A	0.545104	TCGA-L5-A88S-01A	2.195644
TCGA-LN-A49U-01A	0.669384	TCGA-L5-A88Z-01A	1.592383
TCGA-LN-A49X-01A	0.641727	TCGA-L5-A8NK-01A	1.234266
TCGA-LN-A49Y-01A	0.984919	TCGA-LN-A49M-01A	1.880819
TCGA-LN-A4A1-01A	1.065067	TCGA-LN-A49O-01A	1.853437
TCGA-LN-A4A3-01A	0.313926	TCGA-LN-A49P-01A	1.180956
TCGA-LN-A4A4-01A	1.080062	TCGA-LN-A49W-01A	5.693379
TCGA-LN-A4A8-01A	0.228449	TCGA-LN-A4A5-01A	2.106424
TCGA-LN-A5U5-01A	0.831791	TCGA-LN-A4A9-01A	3.995509
TCGA-LN-A5U7-01A	1.128944	TCGA-LN-A4MQ-01A	1.424306
TCGA-LN-A7HV-01A	0.400431	TCGA-LN-A5U6-01A	1.131791
TCGA-LN-A7HW-01A	0.373354	TCGA-LN-A7HX-01A	3.145394
TCGA-LN-A7HY-01A	0.433279	TCGA-LN-A7HZ-01A	2.266462
TCGA-LN-A8HZ-01A	0.568412	TCGA-LN-A9FO-01A	1.956999
TCGA-LN-A8I0-01A	0.245564	TCGA-LN-A9FP-01A	1.748654
TCGA-LN-A8I1-01A	0.765414	TCGA-LN-A9FQ-01A	5.487214
TCGA-LN-A9FR-01A	0.873045	TCGA-V5-A7RC-01B	9.256495
TCGA-S8-A6BW-01A	1.077954	TCGA-V5-A7RC-06A	29.19284
TCGA-V5-AASV-01A	0.160643	TCGA-VR-A8EP-01A	1.41454
TCGA-VR-A8EO-01A	0.042687	TCGA-VR-A8ER-01A	1.817695
TCGA-VR-A8EX-01A	0.778094	TCGA-VR-A8ET-01A	41.92
TCGA-VR-A8EY-01A	0.14391	TCGA-VR-A8EU-01A	2.327484
TCGA-VR-A8Q7-01A	0.38055	TCGA-VR-A8EW-01A	1.293082
TCGA-XP-A8T6-01A	0.616861	TCGA-VR-A8EZ-01A	7.495614
TCGA-Z6-A8JD-01A	0.489117	TCGA-VR-AA4G-01A	3.417357
TCGA-Z6-A8JE-01A	0.295529	TCGA-VR-AA7I-01A	1.554063
TCGA-Z6-A9VB-01A	0.509128	TCGA-XP-A8T8-01A	1.323147
TCGA-Z6-AAPN-01A	0.444947		
